# Ether vs. Chloroform

**Published:** 1882-08

**Authors:** Pedgin Teale

**Affiliations:** Surgeon to the General Infirmary at Leeds.


### ARTICLE VI.

### *Ether vs. Chloroform.*

BY DR. PEDGIN TEALE, SURGEON TO THE GENERAL INFIRMARY AT LEEDS.

It is confessedly difficult, perhaps even impossible, to settle, by statistics, the question of the relative danger of these two anæsthetics ; chiefly for the reason that, while we know pretty nearly how many deaths from each agent occur during the year, we have not the means of ascertaining the relative proportions of the cases in which each anæsthetic has been used.

Such being the case, it may be worth while to record the opinions of those who, having for a great number of years had experience of chloroform, have also for many years (in my own case, more than six years), almost abandoned it in favor of ether. I wish, therefore, to tender my conclusions for what they are worth, based as they are, upon what I have seen in the practice of my colleagues and myself at the Leeds Infirmary, and upon my experience of anæsthetics in my private practice. My conclusions are as follows :

1. Ether, properly administered, is a much safer anæsthetic than chloroform. So much safer do I believe it to be, that I counsel every surgeon whom I can influence in the matter to study the method of its administration, and, to let ether take the place of chloroform. The exceptions I make in favor of chloroform are : in infants, in patients subject to asthma or chronic bronchitis, and also, perhaps, in cases of abdominal obstruction, with difficult breathing in which an operation has to be performed.

2, When many operations have to be done in rapid succession, to use ether is a great economy of time. A good " etherist " can get most patients under its influence in from one and a half to two minutes, whereas, in my experience, chloroform must be given from six to fifteen minutes before an operation can be commenced. I am aware that chlorotormists trained in Edinburgh usually administer chloroform more rapidly than those trained in English hospitals.

3. A patient under the influence of ether is far more passive, and, therefore, in a more convenient condition for operation, than one under chloroform. As soon as the effect of chloroform is passing off, the patient becomes as a rule, and often very suddenly, very sensitive to pain; whereas, in the case of ether, especially if the patient has been kept for some time under its influence, the return of sensibility to pain is very slow. In fact, a patient may become so far conscious as to converse with the surgeon while stitches are being made in the wound, and at the same time be entirely unconscious of pain. This was not my experience of chloroform.

4. When ether is administered without food on the stomach, troublesome sickness is very rare.

5. In using ether, the safety and comfort of the patient, the rapidity of the anæsthesia, and the convenience of the surgeon in operating, depend very directly upon the method of administration employed, and the manner in which the administrator does his work.

6. There are good methods of administration of ether and bad methods, and there are good and bad "etherists." The varying opinions of the value ether which prevail in the profession probably depend very directly upon the varying methods and manners of administration.

7. It is a bad method to give "ether on a towel," as first taught us by Dr. Joy Jeffreys, to whom England is deeply indebted for his successful crusade in favor of ether. This involves a great waste of ether, ten to twenty ounces being required. The patient's lungs are chilled, and bronchial *rales*, struggling, and maniacal excitement not unfrequently result. I, along with my colleagues, commenced ether under this system as a duty, and by no means an agreeable one, and held on doubtfully when I suspected that some patients probably died from the effects of the chilling of the lungs.

8. It is a bad method te give ether with the American basket-work frame, which, though not much better than the towel, served a good purpose as a step to better things.

9. The good methods are those in which the patient breathes over ether into an india rubber bag, a method, I believe, introduced into practice by Dr. Ormsby, of Dublin, and carried to further perfection by Mr. Clover. In this method, the patient breathes the same air over and over again for six or eight times, thereby, economizing the heat of the air passages, economizing ether, and enhancing the effect of the ether by partial asphyxia. My experience of the use of Clover's smaller inhaler, under good management, is this: (*a*) A patient can generally be ready for operation in a minute and a half, sometimes in less than a minute; (*b*) There is rarely any struggling; (*c*) Noisy excitement hardly ever occurs—perhaps, in my practice once in a hundred times; (*d*) *Rales* in the trachea are but seldom heard; (*e*) Instead of six or eight ounces of ether being used in a short operation, and sixteen to twenty in one lasting an hour or an hour and a half, half an ounce or less suffices for a short operation (such as "sphincter-stretching" or "iridectomy"), and two to three ounces for an operation of an hour's duration, such as colotomy or ex cision of a joint.

10. The administration of ether by inferior methods is still too common, and was until recently prevalent in some of our larger hospitals.

11. Even with Ormsby's or Clover's inhalers, there is an infinite variety of skill in different etherists.

12. In order to become a good etherist, the administrator must *study how* to give either, must watch the patient *attentively* while giving it, and during the earlier inhalations must very carefully and *studiously adjust* the unæsthetic to the sensations of the patient.

13. A careful, attentive, student, with tact, and not hard and unfeeling, can easily and in a short time be taught to give ether properly.

Since the adoption of Clover's inhaler, I have had singular freedom from anxiety about my anæsthetics—far greater freedom than in the previous period, when I had to depend upon chloroform.

Finally, I would say that this favorable opinion of ether is based upon my experience of its use by a series of very able administrators—some in the Leeds Infirmary, others while acting as my private clinical assistants. Speaking as a looker-on, rather than as an administrator, I should say that the chief points in the right administration of ether are: first, to overcome the nervous dread of the patient by applying the mouthpiece only; then to turn on the ether gently, until the glottis becomes tolerant and the patient is slightly unconscious; lastly, to complete the anæsthesia rapily. In advising beginners, I compare the regulation of the quantity of ether to the "curve of harmonic progression."—*British Med. Journal.*

